# Advancements in Coronary Bifurcation Stenting Techniques: Insights From Computational and Bench Testing Studies

**DOI:** 10.1002/cnm.70000

**Published:** 2025-03-14

**Authors:** Andrea Colombo, Claudio Chiastra, Diego Gallo, Poay Huan Loh, Socrates Dokos, Mingzi Zhang, Hamed Keramati, Dario Carbonaro, Francesco Migliavacca, Tapabrata Ray, Nigel Jepson, Susann Beier

**Affiliations:** ^1^ Sydney Vascular Modelling Group, School of Mechanical and Manufacturing Engineering University of New South Wales Sydney New South Wales Australia; ^2^ PolitoBIOMed Lab, Department of Mechanical and Aerospace Engineering Politecnico di Torino Turin Italy; ^3^ Department of Cardiology, National University Heart Centre National University Health System Singapore Singapore; ^4^ Yong Loo Lin School of Medicine National University of Singapore Singapore Singapore; ^5^ Graduate School of Biomedical Engineering University of New South Wales Sydney New South Wales Australia; ^6^ Department of Chemistry, Material and Chemical Engineering Politecnico di Milano Milan Italy; ^7^ School of Engineering and Technology University of New South Wales Canberra Australian Capital Territory Australia; ^8^ Prince of Wales Clinical School of Medicine University of New South Wales Sydney New South Wales Australia; ^9^ Department of Cardiology Prince of Wales Hospital Sydney New South Wales Australia

**Keywords:** bifurcation, computational fluid dynamics, finite element analysis, multi‐objective optimization, percutaneous coronary intervention, stent

## Abstract

Coronary bifurcation lesions present complex challenges in interventional cardiology, necessitating effective stenting techniques to achieve optimal results. This literature review comprehensively examines the application of computational and bench testing methods in coronary bifurcation stenting, offering insights into procedural aspects, stent design considerations, and patient‐specific characteristics. Structural mechanics finite element analysis, computational fluid dynamics, and multi‐objective optimization are valuable tools for evaluating stenting strategies, including provisional side branch stenting and two‐stenting techniques. We highlight the impact of procedural factors, such as balloon positioning and rewiring techniques, and stent design features on the outcome of percutaneous coronary interventions with stents. We discuss the importance of patient‐specific characteristics in deployment strategies, such as bifurcation angle and plaque properties. This understanding informs present and future research and clinical practice on bifurcation stenting. Computational simulations are a continuously maturing advance that has significantly enhanced stenting devices and techniques for coronary bifurcation lesions over the years. However, the accurate account of patient‐specific vessel and lesion characteristics, both in terms of anatomical and accurate physiological behavior, and their large variation between patients, remains a significant challenge in the field. In this context, advancements in multi‐objective optimization offer significant opportunities for refining stent design and procedural practices.

## Graphical abstract

This review article examines computational and bench testing approaches in coronary bifurcation stenting, focusing on procedural strategies, stent design, and patient‐specific factors. Key findings highlight the potential of computational methods, such as finite element analysis and computational fluid dynamics, to assess stent performance, optimize deployment strategies, and guide personalized treatment decisions. By identifying current gaps and emerging trends, such as multi‐objective optimization, this work provides a roadmap for advancing stenting techniques and improving patient outcomes.

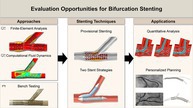

## 1 Introduction

Coronary artery disease (CAD) significantly reduces the quality of life with debilitating and even fatal consequences for more than 154 million people worldwide every year [1, 2]. CAD leads to a partial or total blockage of the coronary lumen, resulting in reduced blood flow and inadequate oxygen supply to the heart [1]. The genesis of CAD is predominantly associated with the development of atherosclerosis, a plaque build‐up in the arterial wall arising from long‐term endothelial homeostasis alteration [3, 4]. Various risk factors intricately contribute to atherosclerosis, encompassing systemic, biological, and biomechanical aspects [5, 6]. Systemic factors, such as hypertension, diabetes, and obesity, can activate inflammation mediators and disrupt endothelium cells [6, 7]. Biological factors, including cholesterol levels and genetic predisposition, further influence disease susceptibility [6]. Biomechanical hemodynamic factors are increasingly recognized for their role in both onset and atherosclerotic disease progression [6, 8]. However, these are not routinely evaluated in clinical practice at present. Indeed, abnormal endothelial shear stress (ESS), sensed by transmembrane flow‐dependent channels [6], is primarily recognized for its role in the initiation and progression of atherosclerotic lesions [5, 8].

Percutaneous coronary intervention (PCI) with stents is the preferred treatment option to revascularize severely stenosed coronary arteries, whereby a balloon‐expandable stent is implanted to restore the arterial lumen patency. Whilst advancements in stent technology have progressed over time, from bare metallic stents to ultra‐thin drug‐eluting stents (DES), their implantation can disrupt vessel geometry and flow conditions, regardless of their design [9, 10, 11], with abnormal ESS [12, 13]. ESS and, in general, hemodynamic disturbances are further exacerbated by stent malapposition [14], which can occur after deployment. Stent insertion can also increase arterial stresses, which are an indicator of arterial damage [15, 16, 17].

Furthermore, strong evidence exists that adverse flow conditions and arterial damage have a direct effect on in‐stent restenosis (ISR), triggered through cellular responses, inflammation, and neointimal proliferation processes within the stented region [18, 19, 20]. Additionally, ESS is a critical factor in various mechanisms contributing to in‐stent thrombosis, further emphasizing its significance in coronary bifurcation stenting [21, 22, 23].

PCI in coronary bifurcation lesions represents 15%–20% of all stenting procedures [24, 25], with nearly half of these interventions targeting the left main (LM) [26]. These are considered especially challenging [27] due to complex branching shape and hemodynamics [11, 18], resulting in worse clinical outcomes with respect to non‐bifurcating regions, with rates of adverse events being 19% versus 12%, respectively [28, 29]. As a result, the management of complex bifurcation lesions is of significant interest, but despite numerous clinical and computational investigations it remains a topic of ongoing debate among experts [30]. A one‐stent approach, known as provisional side branch (PSB) technique, has demonstrated superior outcomes in simple lesions [30] and thus is generally preferred in clinical practice. Complex bifurcation lesions are marked by significant side branch (SB) plaque and/or challenging anatomy, such as extreme bifurcation angles, large diameter variations, or long, wide‐spread and severe lesions. Those lesions, which occur in almost a third of all patients [31], might require further consideration and generally necessitate a two‐stent approach [32]. In these scenarios, the lack of understanding of the role of vessel and lesion characteristics in bifurcation stenting strategies becomes deleterious [33, 34].

This review examines the current state of stenting approaches for bifurcation lesions and explores the influence of procedural aspects, stent design and patient‐specific characteristics on the outcome of PCI with stents. Key methods are explored which aid the exploration of bifurcation stenting outside of clinical trials. Specifically, computational methods, including structural mechanics finite element analysis (FEA), computational fluid dynamics (CFD), and fluid–structure interaction (FSI), are first described for the analysis of stent deployment and bifurcation hemodynamics. Then, bench testing methods are introduced and reviewed, before discussing subsequent emerging multi‐objective optimization (MOO) efforts in the context of coronary stents. The review concludes by summarizing key findings, discussing the current state of the field, and identifying potential opportunities for guiding clinical intervention strategies for bifurcation lesions.

## 2 Coronary Bifurcation Stenting Strategies and Their Ongoing Clinical Debates

PSB involves wiring both the main vessel (MV) and SB, deploying in the MV a single stent sized to the distal MV, jailing the SB wire, and performing the proximal optimization technique (POT) [35]. Additional SB intervention, including a rescue stent, is only performed if the SB is at risk of occlusion [36] (Data S1). PSB remains the preferred and most common strategy [37], even for complex bifurcation lesions if the SB diseased region is shorter than 10 mm [28, 37, 38].

**FIGURE 1:**
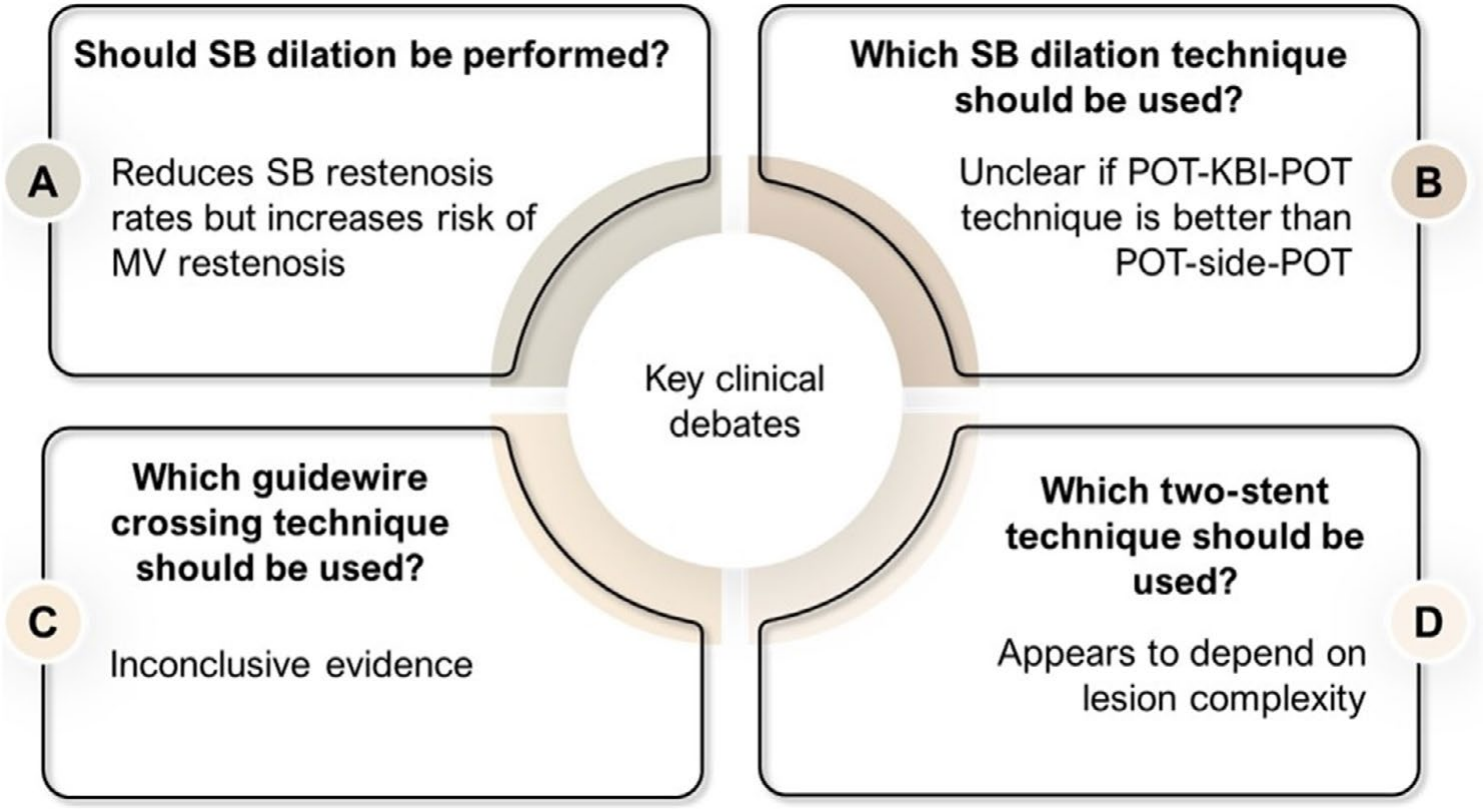
Key clinical debates in coronary bifurcation stenting. (A) Deciding not to perform KBI after stenting will leave struts across the SB ostium and can enhance flow disturbance and potentially lead to thrombotic or restenotic events, but dilation of the SB might lead to unfavorable outcomes. (B) There is no conclusive evidence of superiority of SB dilation technique, including methods of POT‐KBI‐POT or POT‐side‐POT. (C) There are various strategies aimed at mitigating SB compromise during stenting, but there is still a lack of extensive clinical data comparing these methods. (D) There is conflicting evidence around which two‐stent technique leads to better clinical outcomes. KBI: kissing balloon inflation; MV: main vessel; POT: proximal optimization technique; SB: side branch.

A significant area of debate in coronary bifurcation treatment centers around if kissing balloon inflation (KBI) should be routinely used after MV stenting to reduce the risk of SB thrombosis [39] (Figure 1). KBI consists in the simultaneous inflation of two balloons in both the MV and SB [40]. KBI effectiveness showed conflicted results, demonstrating both a reduction of SB restenosis rates [41], and concerns regarding the increased risk of MV restenosis [41, 42]. However, balloons with a short overlap (< 3 mm) were shown to decrease target lesion revascularization with respect to a longer overlap [43]. Additionally, bench testing data showed that long balloon overlap when performing KBI is associated with increased MV stent deformation, particularly with wider bifurcation angles when compared to minimal balloon overlap [40]. KBI helps clear stent struts from the SB ostium, but it may also cause overexpansion of the stent, resulting in increased arterial damage and ellipticity [44]. To mitigate these issues, POT can be employed. POT involves inflating a short balloon to ensure proper apposition of the stent to the proximal vessel wall and to restore the natural branching pattern at the bifurcation site [25, 28, 45]. These diverse outcomes show the complexity in choosing the intervention strategy and the necessity for patient‐specific treatment strategies [46]. Another core debate revolves around the use of the POT‐side‐POT strategy (Figure 1), involving sequential balloon inflations in the proximal MV, SB, and then the proximal MV again. Despite its common usage attributed to lower risks of adverse outcomes, this strategy presents a concern regarding stent distortion, unlike for the POT‐KBI‐POT technique [44, 47]. Furthermore, a critical aspect of the clinical debate centers on the implementation of various guidewire crossing strategies to address SB compromise during stenting (Figure 1). SB compromise refers to the unintentional blockage or narrowing of the SB following MV stenting and can result from the displacement of the carina or the plaque [48]. A detailed description of those techniques is reported elsewhere [49]. Polymer‐coated guidewires have shown more resistance to retrieval damage and quicker SB crossing compared to non–polymer‐coated wires, which are more susceptible to damage and can lead to procedural complications [50]. Although progress has been made in understanding SB dilatation methods, especially regarding guidewire strategies, further clinical data are needed to comprehensively compare their long‐term outcomes (Figure 1).

Elective two‐stenting techniques, which involve the pre‐planned use of two stents in the treatment of the lesions [30, 51], are preferred in the case of complex bifurcation lesions characterized by SB with large caliber and diffuse plaque [30, 32]. The specific stent deployment strategy in the SB and subsequent main stent implant can vary significantly, especially in the way the stents overlap. Most common techniques include the T, T‐and‐protrusion (TAP), culotte, and crush variations such as double‐kissing crush (DKC) and nano‐crush [24, 28, 52] (Data S2–S4).

Whilst DKC was proven to be superior to PSB in complex lesions [53, 54, 55], there is only limited literature on the comparisons between two‐stenting techniques. Specifically, DKC showed better outcomes, in terms of reduction in target lesion revascularization, cardiac death, and in‐stent thrombosis, than culotte, crush, and T/TAP techniques, especially in LM bifurcations [38, 56, 57, 58]. The classic crush technique showed no difference compared to the culotte technique for both short (< 6 months) [59] and long‐term (> 12 months) outcomes [60, 61]. These findings may be affected by the bifurcation angle [28], whereby acute bifurcation angles lead to a greater quantity of metal at the carina, and obtuse bifurcation angles to incomplete ostium strut coverage [62]. In fact, the culotte technique appears suitable for acute angles < 70° [63], and the T‐technique for angles of approximately 90° only to assure complete SB ostium coverage [64]. TAP was found suitable for 70°–90° bifurcation angles [28], and DKC was shown to perform well regardless of the bifurcation angle [28]. Besides these studies, research to date has not yet determined overall which of these two‐stenting technique variations is preferable in which scenario, with clear guidelines still missing. This is likely due to a large variation of patient‐specific anatomy and plaque composition, as well as variability in experience and competence of the interventional operator. Australasian clinicians typically prefer the DKC approach, while European counterparts favor the culotte technique. Furthermore, a lack of randomized clinical data compounds the challenges. Hence, there is a clear need for a better understanding of which technique variation may be more suited to a specific lesion characteristic, and how this knowledge may translate into clinical guidance and selection of interventional strategy.

## 3 Methods for Stent Technique Evaluation and Optimization

**FIGURE 2:**
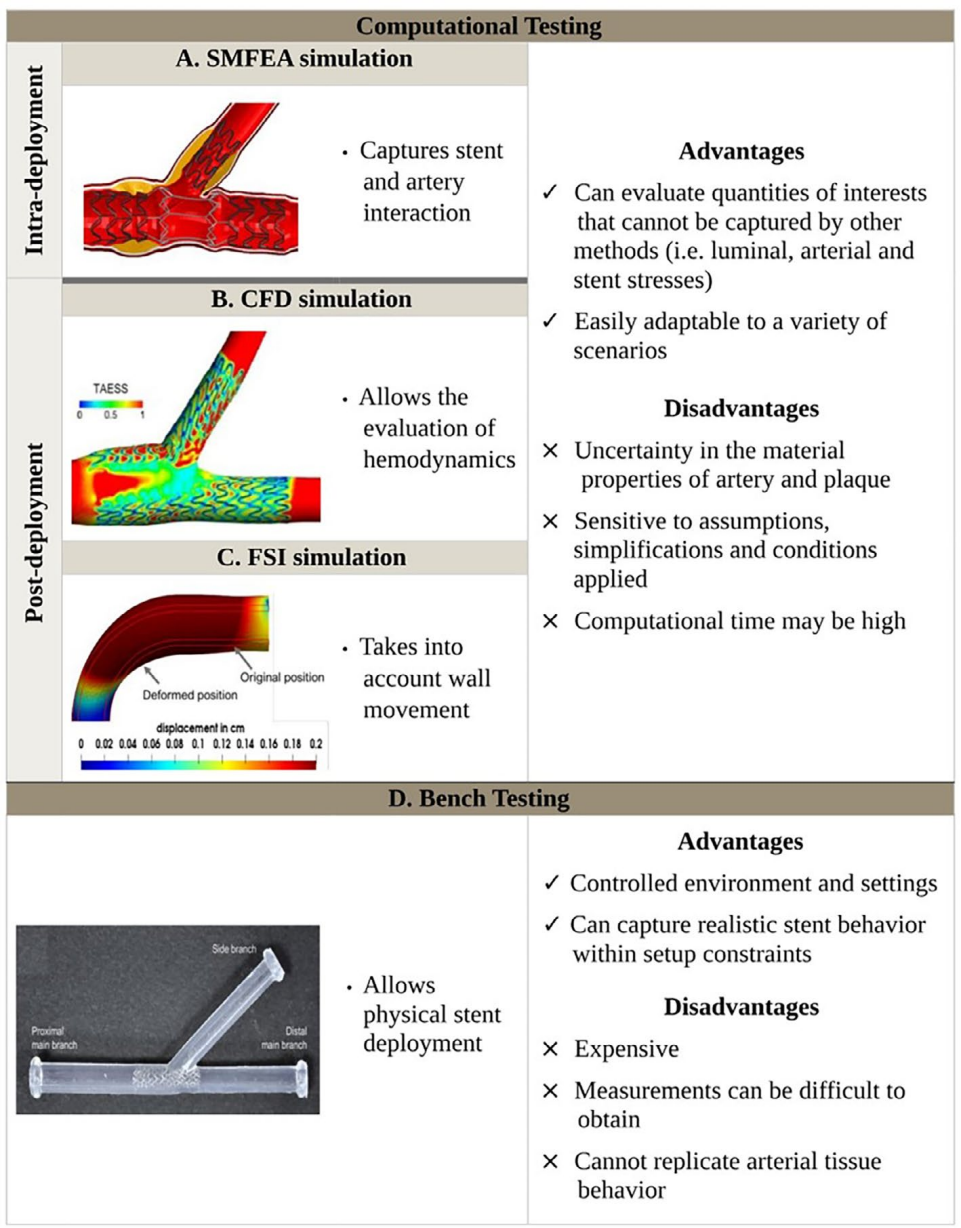
Main methods used to analyze stenting techniques (A) SMFEA models allow the simulation of each step of a stenting technique and calculation of objectives of interest during deployment (adapted with permission from Arokiaraj et al. [66]). (B) CFD models allow the evaluation of the hemodynamics after stent deployment at different time instants of the cardiac cycle (adapted with permission from Morris et al. [67]). (C) FSI simulations combine structural mechanics and fluid dynamics models to take into account arterial wall compliance during the cardiac cycle (adapted with permission from Balzani et al. [68]). (D) Stented phantoms used for in vitro testing (bench testing) (adapted with permission from Chiastra et al. [69]). CFD: computational fluid dynamics; FSI: fluid–structure interaction; SMFEA: structural mechanics finite element analysis; TAESS: time‐averaged endothelial shear stress [70, 71, 72].

Computational methods have become important to understand the complex relationship between stent, arterial wall, and blood flow. Typically, a structural mechanics FEA and/or a blood fluid dynamics CFD simulation is conducted. Structural mechanics FEA allows evaluating the mechanical interplay between the device and the arterial wall during each phase of the stent deployment process through the calculation of stress and strain distributions within the artery and the stent [65] (Figure 2). The CFD approach employs numerical methods to solve the governing equations of fluid motion, commonly used for the analysis of the post‐deployment hemodynamics, including velocity, pressure, and local hemodynamic features [73] (Figure 2). While it is also possible to couple these methods in an FSI analysis, this technique has not been employed in the context of bifurcation stenting strategies but only in straight coronary artery stenting [74, 75]. Even though computational methods may be time intensive, they can be readily applied to a variety of cases, and are easily reproducible and controlled, thus providing detailed insights that cannot be reliably captured with bench efforts or via clinical trials [70]. Computational analysis helps clinical understanding by revealing key aspects of stent behavior and hemodynamics, complementing intracoronary imaging techniques such as intravascular ultrasound and optical coherence tomography. By simulating various deployment scenarios, computational methods allow for predicting stent‐related complications, such as malapposition, and identify potential areas of disturbed flow that may contribute to thrombosis or ISR. Furthermore, modeling the effects of different deployment techniques helps create tailored treatment strategies to improve the success rates of bifurcation stenting procedures. Imaging and computational modeling are complementary approaches. While imaging data should be used to validate the computational models, the models can also be used to investigate the parameters that are difficult (if not impossible) to measure using intracoronary imaging. In contrast, bench testing involves the implant of stent(s) in a bifurcation replica or phantom (Figure 2) and is often used in conjunction with CFD simulations. While useful for evaluating stenting strategies, it lacks the ability to fully capture the nuances of stent deployment and hemodynamics. All methods and their critical studies are discussed as follows.

### 3.1 Computational Simulations

Geometries of idealized coronary bifurcations and plaque are usually generated through computer‐aided design software using clinical data obtained from the literature. Patient‐specific coronary bifurcations are instead reconstructed from the combination of optical coherence tomography, intravascular ultrasound and/or computed tomography (CT) coronary angiography images [76, 77], together with the deployed stent [78, 79]. When stent virtual geometries are not available from stent manufacturers, the geometries are usually reconstructed from micro‐CT images or from the manufacturer's datasheet [80, 81, 82, 83, 84], while balloon geometries are usually generated to resemble commercial devices used in clinical practice [85, 86, 87].

The assumptions made and simplifications applied to develop a computational model may significantly affect the accuracy of the computed results. A key challenge remains the accurate capture of the arterial wall and plaque properties; that is, shape, composition and their mechanical properties [88]. For the hemodynamic description, blood is typically assumed as an incompressible fluid with constant density and non‐Newtonian behavior, necessary when simulating stented regions to accurately capture the near‐stent‐shear changes [73]. Choosing appropriate inlet and outlet boundary conditions is an important consideration, greatly affecting the accuracy of the model. Inlet boundary condition was often implemented as a pulsatile blood flow set as a paraboloid‐shaped velocity profile, with average flow rates obtained from literature [12, 82, 89, 90, 91].

Computed outcomes from both CFD and structural mechanics FEA simulations are important for assessing stent performance and optimizing clinical outcomes in coronary bifurcation stenting. Structural mechanics FEA allows the assessment of stent expansion, strut malapposition, lumen gain, SB ostium clearance, stent foreshortening, dogboning, and flexibility [92]. Meanwhile, CFD outcomes include the ESS and its cardiac cycle time‐averaged endothelial shear stress (TAESS), measuring the blood's shear stress force imposed onto the endothelium, the oscillatory shear index (OSI), a metric that quantifies the directional changes of ESS [13], and the relative residence time (RRT), which represents the duration of a blood particle's residence at the endothelium [13].

### 3.2 Bench Testing

Bench testing is an effective in vitro method to investigate the feasibility and efficacy of different stenting strategies for bifurcation lesions (Figure 2) and involves the creation of a physical bifurcation phantom [70, 71]. Originally, phantoms were created from casting and molding techniques, offering the possibility of creating transparent models and phantoms with varying levels of rigidity, softness, or resistance [71]. This was achieved by using materials such as PMDS, MoldStar 15, EcoFlex 00‐30, and DragonSkin [71]. The introduction of modern techniques, such as 3D printing, has changed how phantoms are currently made. Several types of 3D printing technologies are available today, each with its own characteristics. These include stereolithography (SLA), material jetting (MJ), fused deposition modeling (FDM), and selective laser sintering (SLS).

Casting and molding techniques can be cost‐effective on a large scale due to lower unit costs once molds are created. However, higher costs may be involved in research settings requiring a limited number of phantoms or patient‐specific geometries, where 3D printing offers faster and more economical solutions [71]. Additionally, 3D printing is particularly advantageous for creating complex geometries and patient‐specific phantoms due to its efficiency and customization capabilities [71].

After stent deployment within the phantom, micro‐CT reconstruction is often used to visualize and evaluate the stented geometry [93], providing valuable insights into stent expansion and (mal)apposition, and strut deformation [93]. One of the key advantages of bench testing is the ability to control and standardize the testing environment, eliminating the influence of anatomic variability and the uncertainties in the mechanical properties. This aspect facilitates the evaluation of different stenting strategies and stent types, including the performance of ultra‐thin stents compared to thicker stents. However, there are limitations to benchtop testing. Specifically, the presence of plaque is often neglected [70, 81, 94, 95, 96], which inevitably affects the behavior of the stent and thus results in a model with limited applicability to clinical scenarios. In addition, despite some reports on the fabrication of multilayered phantoms that closely resemble the properties of both healthy [97], and diseased [98] coronary arteries, replicating the complex patient‐specific geometry of a bifurcation remains challenging. This includes accurately representing the compliance of carinas, which are usually made too rigid compared to in vivo [70], thus not allowing the carina to move after stent deployment. Hence, directing research efforts toward identifying material and manufacturing solutions is crucial in creating realistic bifurcation phantoms [70].

### 3.3 Multi‐Objective Optimization

Successful stenting necessitates a meticulous consideration of multiple, often conflicting, conditions simultaneously. The intricate interplay between various structural mechanics and hemodynamic factors poses a challenge. To effectively address these complexities, MOO emerges as a powerful tool. MOO is a mathematical approach employed to tackle complex problems involving multiple conflicting objectives. Rather than optimizing one objective while neglecting others, MOO aims to identify solutions with the best trade‐offs between different objectives [99]. These solutions, known as non‐dominated solutions, perform optimally in one objective without compromising performance in other objectives [99]. From this set of non‐dominated solutions, the most suitable option can be selected. For instance, in optimizing the thickness of a stent strut, MOO was used to determine the ideal balance between radial support, flexibility, and blood flow requirements [100].

When dealing with numerical simulations such as those involved in coronary bifurcation stenting, evaluating the objective function for every design point in the search space can be time and resource‐intensive, and thus, surrogate models are often coupled with MOO methods [99]. MOO used to explore improved stent design possibilities has been successful for the optimization of stent drug release [101], stent design type [102], and optimization across stent design classes [100]. However, to the best of our knowledge, MOO has only been applied once to optimize stenting strategies [103]. Other MOO studies have been applied to stent design only, and their approaches, concepts and findings can infer conclusions relevant to stent strategy research.

While MOO studies typically excluded experimental data due to practical constraints, integrating bench testing could represent a significant advancement in this field. By experimentally informing surrogate models, the need for validation inherent in computational simulations can be avoided, thereby reducing the uncertainties arising from model assumptions. Although yet unexplored in the context of stenting techniques, the successful application of this approach in other domains suggests its feasibility and potential impact [104, 105].

## 4 Stenting Evaluation and Optimization Findings

### 4.1 Consideration of Single‐Stent or Provisional Side Branch Techniques

**FIGURE 3:**
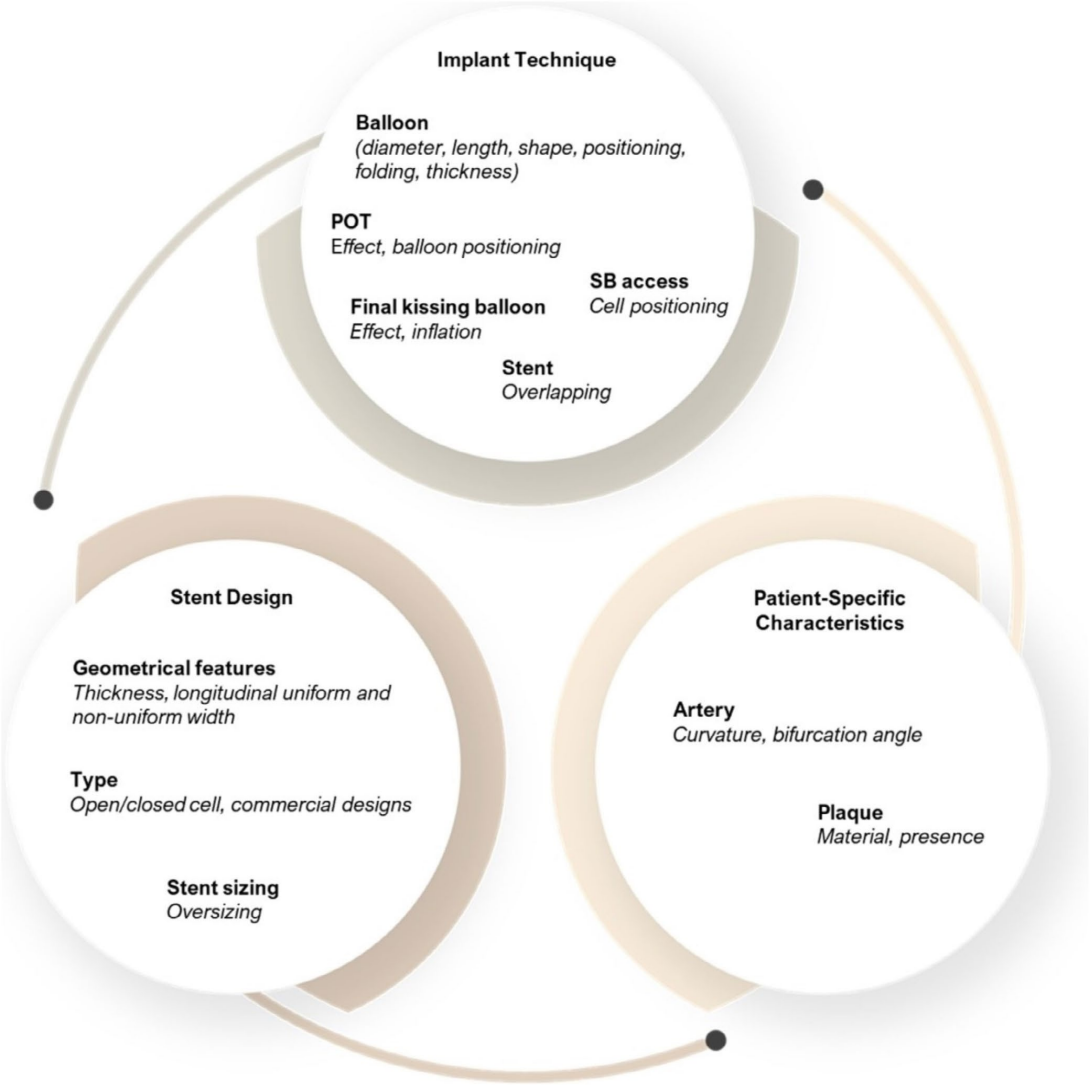
Key factors of PSB stenting success studied to date: procedural aspect, stent design, and patient‐specific characteristics. POT: proximal optimization technique; PSB: provisional side branch; SB: side branch.

Computational methods to study the key success factors of PSB (Figure 3) have been widely employed (Table 1) (Data S5). Idealized geometries are commonly used, and plaque has been considered in about half the literature to date. Plaque geometry is usually idealized, and its material properties were mostly represented using an isotropic hyperelastic model coupled with perfect plasticity [48, 80, 85, 89, 90, 113]. Isotropic hyperelastic models were commonly used for describing the material behavior of the arterial wall [80, 82, 84, 112], and one study replicated arterial anisotropic behavior, achieving a good correlation with experimental data [86]. Balloon geometries studied to date commonly resembled commercially available devices used in clinical practice. Their material properties were assumed to be isotropic and linear elastic and were calibrated by comparing them with the pressure‐diameter relations provided by the manufacturers [48, 114]. Balloon extremities are commonly radially constrained while leaving circumferential and longitudinal directions free [80, 89]. After crimping the stent, accomplished via a rigid surface controlled by a radial displacement boundary condition, procedural steps for PSB stenting are simulated according to the recommended protocols. This includes bending balloons by applying rotations to the extremities, marking the KBI step, and facilitating insertion into the SB [80, 89]. Notably, interaction formulations varied, with frictional contact [107], or frictionless contact [112] being utilized in the models.

**TABLE 1:** Comparison of computational and bench testing studies investigating PSB in coronary artery bifurcations.

Key factor	Focus	Type	Coronary bifurcation model			Reference
			Geometry (*n*)	Wall	Plaque (material)	
Implant technique	Comparison of side branch accesses and final kissing balloon vs. POT as the final step	SMFEA	I (1)	Three layers, isotropic hyperelastic	Yes (isotropic hyperelastic with plasticity)	Gastaldi et al. [80]
	KBI technique normal vs. tapered balloon	SMFEA + CFD	I (1)	Three layers, isotropic hyperelastic	Yes (isotropic hyperelastic with plasticity)	Morlacchi et al. [89]
	POT‐side‐POT vs. KBI	BT + SMFEA + CFD	I (1)	Three layers, hyperelastic	No	Foin et al. [81]
	Proximal vs. distal side branch access	SMFEA + CFD	I (1)	Three layers, isotropic hyperelastic	No	Chiastra et al. [82]
	PSB kissing balloon	SMFEA + CFD	I (1)	Not provided	No	Chen et al. [106]
	Proximal optimization of balloon positions	SMFEA	PS (5)	Three layers, isotropic hyperelastic	Yes (homogeneous calcification agglomerate)	Rigatelli et al. [83]
	Effect of balloon and stent‐catheter assembly parameters on the stent deployment	SMFEA	I (1)	One layer, Mooney–Rivlin hyperelastic	No	Rahinj et al. [107]
	Overlapping stents	SMFEA + CFD	PS (2)	One layer, hyperelastic	Yes (isotropic hyperelastic with plasticity)	Chiastra et al. [90]
	POT‐side‐POT	BT	I (40)	Polyvinyl chloride	No	Derimay et al. [108]
Stent design	PSB outcomes with different stent designs	SMFEA	I (1)	Silicone	No	Burzotta et al. [109]
	Stent oversizing	SMFEA + CFD	PS (1)	Three layers	Yes (not provided)	Mortier et al. [110]
	ESS in BRS	CFD	PS (1)	Not provided	No	Migliori et al. [12]
	ESS topological skeleton changes after stenting	SMFEA + CFD	PS (3)	One layer, hyperelastic	No	Chiastra et al. [91]
PS characteristics	Plaque presence impact on hemodynamics	SMFEA + CFD	I (1)	Not provided	Yes (Not provided)	Chen et al. [111]
Implant technique + Stent design	Impact of stent design and side branch access on final strut apposition	BT + CFD	I (1)	Not provided	No	Foin et al. [95]
	Stent design and final KBI strategies	SMFEA	I (3)	Three layers, isotropic hyperelastic	Yes (isotropic hyperelastic)	Mortier et al. [84]
	Stent platforms and balloon sizes	SMFEA	I (1)	One layer, homogeneous isotropic hyperelastic	No	Mortier et al. [112]
Implant technique + PS characteristics	Bifurcation angle, plaque composition, and balloon length	SMFEA	I (8)	Three layers, homogeneous isotropic hyperelastic	Yes (Neo‐Hookean model with plasticity)	Iannaccone et al. [48]
Stent design + PS characteristics	Stent overlap and stent‐induced vessel straightening	SMFEA	PS (2)	One layer, hyperelastic	Yes (isotropic hyperelastic with plasticity)	Morlacchi et al. [85]
	Stenting in a curved coronary bifurcation	SMFEA	PS (1)	Three layers, anisotropic fiber‐reinforced hyperelastic	No	Mortier et al. [86]
Implant technique + Stent design + PS characteristics	Impact of stent‐induced vessel straightening. Effect of stent design and positioning on malapposition rates	SMFEA + CFD	PS (2)	One layer, isotropic hyperplastic	Yes (isotropic hyperelastic with plasticity)	Chiastra et al. [113]

Abbreviations: BRS: bio‐resorbable scaffolds; BT: bench testing; CFD: computational fluid dynamics; ESS: endothelial shear stress; I: idealized; KBI: kissing balloon inflation; POT: proximal optimization technique; PS: patient‐specific; PSB: provisional side branch; SMFEA: structural mechanics finite element analysis.

Limited studies achieved PSB stent deployment using bench testing [81, 95, 108, 115]. Once the bench implanted stent was digitally reconstructed after imaging, CFD simulations were used to investigate the resulting blood flow disturbances from changes in rewiring position [82], post‐dilation strategies [81, 89, 106], or stent oversizing [110]. Various methodologies have been employed to date (Table 1), but it is important to note that these have typically looked at different aspects of coronary bifurcation stenting techniques, making direct comparisons difficult. However, studies focusing on similar topics generally reach consistent conclusions. For example, regardless of methodological differences, most studies agree that POT is more favorable than KBI as the final PSB step [80, 81].

#### 4.1.1 Implant Technique

Implant techniques engulf all aspects of ballooning including the choice of balloon and deployment strategy; that is position and pressure, and wiring techniques throughout the procedure. In fact, balloons and rewiring are two crucial factors that affect stent deployment, especially in coronary bifurcations [44]. Balloons have been extensively studied [80, 89, 106], being an essential component at every step of the intervention.

The optimal balloon diameter was found to be a trade‐off between the stent element distortion and SB injury [112], indicating that a smaller balloon diameter would reduce SB injury but might not adequately expand the stent at the SB ostium. Non‐uniform stent deployment can occur due to variations in balloon thickness due to manufacturing variations, and eccentric alignment of the stent on the balloon [107]. While exhibiting similar expansion behavior, five‐fold balloons have a smaller diameter after deflation compared with tri‐fold ones, making them easier to retrieve [107]. The ideal balloon length depends on the stent length during deployment [107], with shorter balloons being preferred for POT [48]. Better POT results are also achieved when balloon markers are located at the carina [83]. Computational simulations showed that POT is more effective than final KBI in reducing stent malapposition [81], inducing lower arterial wall stresses and device plastic strains [80]. However, final KBI reduces arterial circumferential stress compared to simple PSB [106], and can be further optimized by using a tapered balloon [89], or non‐simultaneous inflation [84]. Moreover, the positioning of stent deployment can influence the percentage of malapposed struts. In particular, a previous study considering a commercial device implanted in three different locations with respect to the first diagonal branch showed that the malapposition rates decreased in the case of the most distal positioning [113].

In the event of long atherosclerotic plaques in one of the branches, two overlapping stents are often deployed clinically to achieve complete lumen patency [85]. However, both structural mechanics FEA and CFD analyses have confirmed that superimposed layers of struts are not a favorable feature [85, 90]. Indeed, higher stresses in the arterial wall and in the stent (close to the ultimate break stress) are generated in the overlapping region [85], as well as higher areas of atherogenic low ESS [90].

Rewiring into the SB has been considered using both CFD [82] and bench testing [95], showcasing that distal recrossing of the guidewire in open‐cell stents optimizes the final KBI technique, with more favorable ESS and lower malapposition rates [82, 95]. However, when the same research question was approached from a structural perspective using structural mechanics FEA simulations, considering stent and arterial stresses, a central re‐crossing was considered more favorable [80]. This suggests a trade‐off between stent and arterial stresses, favorable ESS, and malapposition rates when aiming to identify an ideal technique when rewiring the SB.

Bench efforts showed that obtaining full strut apposition was unfeasible across stent types, highlighting the limits of conventional stents in achieving optimal deployment [95]. Furthermore, in vitro testing provided insights into the performance of the POT‐side‐POT strategy when applied to polymeric and metallic stents [108]. This technique effectively restored the physiological branching pattern of the bifurcation phantoms, while mitigating malapposition and avoiding stent rupture, a common occurrence with polymeric stents [108]. Particle image velocimetry was also used to experimentally evaluate post‐stenting flow dynamics [115]. Notably, PSB stenting resulted in low recirculation areas, but was associated with high OSI and RRT and low TAESS [115].

#### 4.1.2 Stent Design

The design of a stent directly impacts its mechanical characteristics, which in turn influences the safety of the treatment. The optimization of stent design, independent from the stenting strategy, is commonly conducted through computational simulations. Most research in this field has focused on assessing the impact of various stent design characteristics, such as strut profile [116], connector shapes [117], or combinations thereof [14], whereby the mechanical behaviors of the stent, such as radial recoil, dogboning, foreshortening, flexibility, and risk of arterial damage, were modeled using structural mechanics FEA. Specifically, larger strut widths improve radial strength and resist against recoil, but also increase the risk of vessel damage [116]. Connector shape has a significant impact on flexibility and on the stent and arterial stress [117]. More recently, CFD simulations have highlighted the significance of incorporating flow‐related factors into stent design [100, 118]. Indeed, the adverse impact of certain design characteristics can be alleviated in combination with other design features. For instance, the adverse hemodynamic effects, such as low ESS, resulting from increased strut thickness can be mitigated by larger strut spacing [14].

Despite extensive research in this field, there is limited analysis on stent design in the context of coronary bifurcation stenting. Most studies have focused on stent design for straight vessels as reviewed elsewhere [119, 120, 121], and is outside the scope of this literature review.

Computational simulations typically consider idealized and non‐diseased vessels, and there are limited studies with patient‐ or lesion‐specific models to date [85, 86, 113] (Table 1). Key findings include that thicker‐strutted stents elevated arterial stresses [86], and the use of an oversized stent may reduce malapposition but also increases the likelihood of a carina shift and arterial wall stresses [110]. Interestingly, malapposition is often misconceived as a result of poor deployment only, however malapposition rates are equally affected by stent design, because different stent/strut geometries can interact differently with the non‐uniform coronary wall [113]. Generally, open‐element stents are preferred over closed‐element designs due to their improved SB access [112], and flexibility [86], inducing less arterial damage. Accordingly, only open‐element designs are currently used in clinical practice [122]. There is no apparent difference in malapposition rates and SB access for commercial open‐element stent designs [84, 95, 109]. Interestingly, the number of connectors had no impact on the SB occlusion [84, 95], even though a higher number of connectors is associated with improved stent performance [17] and clinical outcomes [123, 124].

#### 4.1.3 Patient‐Specific Characteristics: Bifurcation Angle, Diameter, Plaque Composition

**FIGURE 4:**
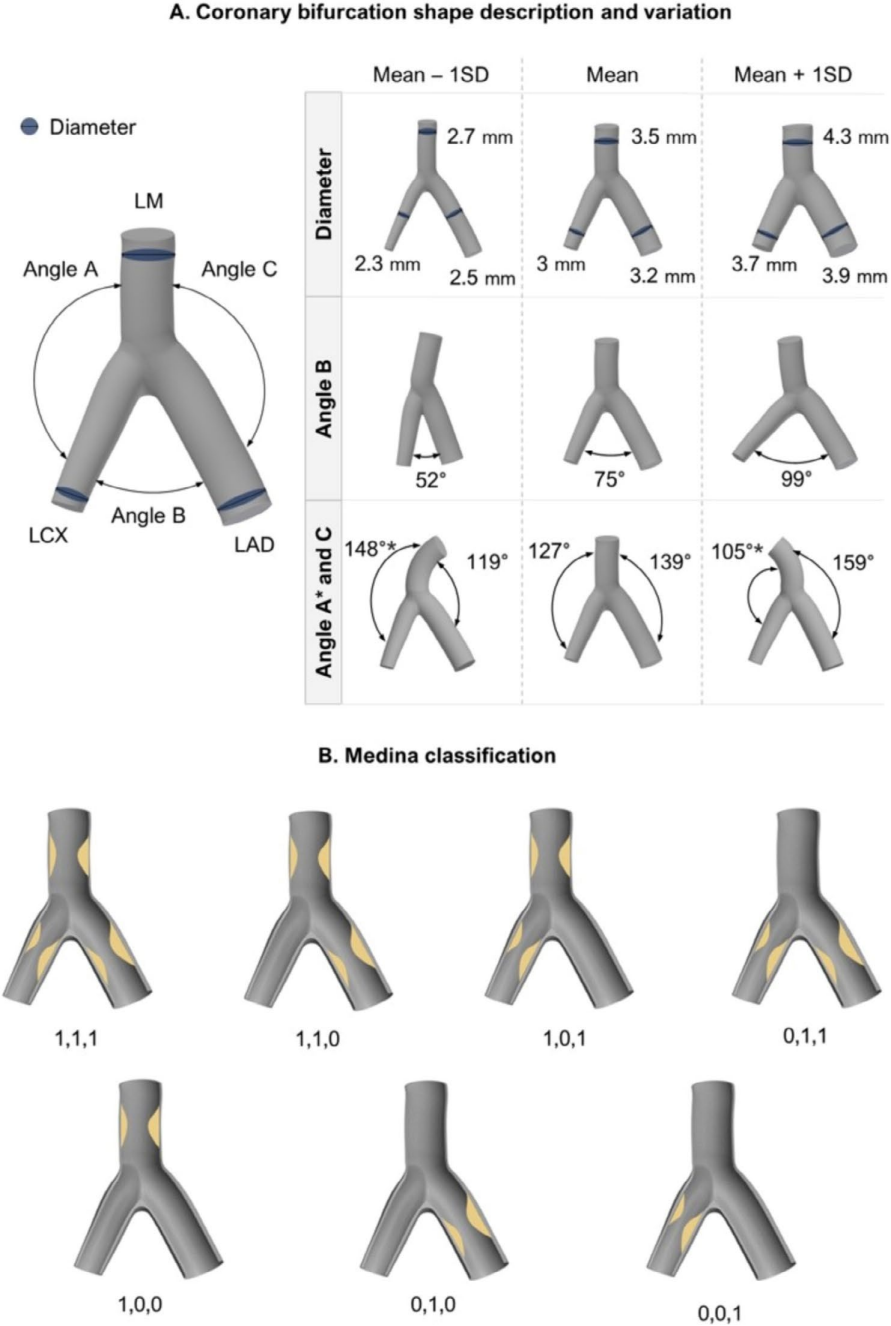
(A) Shape description and variation of a LM bifurcation. The geometry values are obtained from the sum of the mean and 1SD values for the population to describe the range of values for diameters and bifurcation angles [125]. (*) denotes angles A and C varying inversely with respect to the mean, that is, on the left side, angle A corresponds to mean + SD. (B) Medina classification for bifurcation lesions. 0/1 indicates the absence/presence of a stenosis in the bifurcation branch. LM: left main; LAD: left anterior descending; LCX: left circumflex; SD: standard deviation.

Coronary artery characteristics, such as bifurcation angle, vessel caliber, and plaque composition, widely vary among people (Figure 4) [125]. Ethnicity contributes, with the Asian population tending to have overall smaller coronaries compared to the Caucasian population [126]. Disease is commonly classified using the Medina distribution (Figure 4) [127], where Medina 1, 1, 1 is the most common (35.5%), indicating plaque in the MV, dominant daughter vessel and SB—respectively. This is followed by 1, 1, 0 (26.8%) and 0, 1, 0 (10%), with 0, 0, 1 being the least common (3.5%) in Caucasians [128]. Given this wide range of patient‐specific physiological and pathophysiological characteristics, it becomes reasonable to argue that these need to be considered when selecting a stenting strategy. Limited efforts in this pursuit showed that when stenting a curved or tortuous coronary artery, the compliance mismatch between the coronary and the stent causes vessel straightening and may result in an abrupt increase of ESS and arterial stresses at the stent ends [85, 86, 113]. It was further found that the bifurcation angle may impact SB access [48], however, the clinical literature still has no consensus on the role of the bifurcation angle in PSB [129, 130]. When comparing stent deployments with and without plaque in the SB, the presence of disease resulted in more unfavorable ESS and increased arterial damage in the SB [111] than in disease‐free counterparts. This suggests that plaque should be included in virtual models to capture more realistic behavior, as otherwise important physiological effects of ESS and arterial stresses may be underestimated. Indeed, plaque composition affects SB ostial access [48], with lipid and fibrous plaques having almost no effect on SB dimensions due to their soft nature and thus compliance, resulting in minimal resisting effects during stent deployment. However, in the case of calcified plaques, the plaque was pushed toward the SB ostium, resulting in severe SB compromise with important volume and area lumen distortions [48]. Plaque shape and location in the context of PSB stenting have not been evaluated elsewhere.

#### 4.1.4 Multi‐Objective Optimization of Stent Design

**FIGURE 5:**
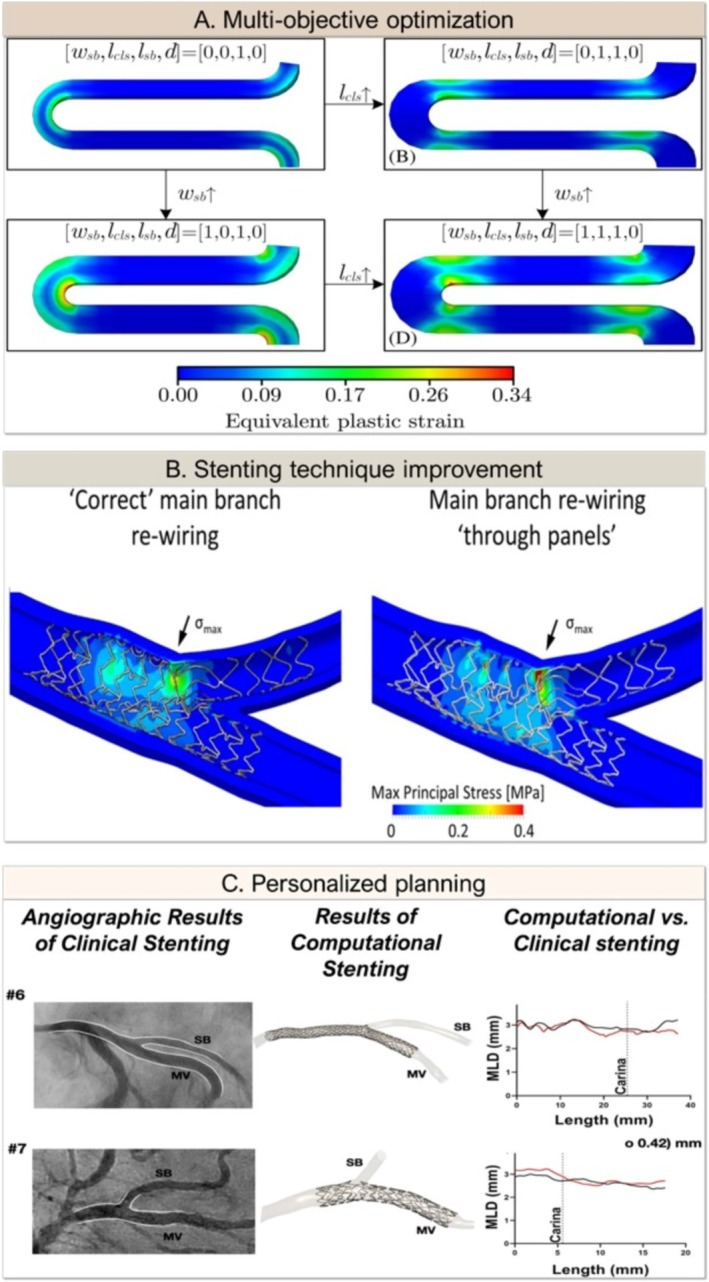
Key frontiers in the field of computational simulation for coronary bifurcation stenting strategies. (A) Different strut designs can be evaluated through multi‐objective optimization. For example, different stress distributions can be obtained and optimized by varying the strut characteristics. Multi‐objective optimization, combined with surrogate models, will enable the improvement of stenting techniques through the simultaneous exploration of diverse scenarios. Image adapted with permission from Ribeiro et al. [131]. (B) Two different SB accesses were compared to assess their impact on stenting outcome. Computational simulations offer a comprehensive approach to improve stenting outcome. Image adapted with permission from Grundeken et al. [114]. (C) When comparing angiographical results and computational stenting, minimal differences were noticed, thus validating computational patient‐specific pre‐clinical planning. Accurate reproduction of in vivo scenarios is achieved through numerical models, opening to the feasibility of reliable, personalized clinical planning. Image adapted with permission from Zhao et al. [132].

Considering multiple objectives simultaneously enables an overall more comprehensive understanding of the often‐interdependent relationship between design variables and objectives. Consequently, MOO techniques are at the frontier of stent design and strategy research to date (Figure 5). There are existing studies that aimed to optimize coronary stent design by considering multiple performance objectives, sometimes with competing objectives [102, 131, 133]. One MOO approach optimized stent deployment by considering balloon pressure and diameter as design variables, suggesting a patient‐specific interventional protocol balancing arterial stress, stent malapposition and volume average drug [103]. However, most MOO was used to improve coronary stent designs for better performance, whereby studies focused predominantly on structural mechanics objectives [17, 102, 134, 135, 136], and limited attention has been paid to the hemodynamic effects [137, 138, 139], while some integrated both structural mechanics and hemodynamics aspects [100, 140]. A limited number of studies explored drug‐eluting aspects [101, 103, 140].

Conflicting mechanical relationships between radial recoil and vascular injury, as well as between bending and longitudinal resistance, were observed [102]. Thin and long strut stents excelled in minimizing vessel injury and bending resistance, but they performed poorly in terms of radial recoil and longitudinal resistance. Interestingly, minimal connector length improved performance across all metrics against the baseline stent design [102], despite connectors being seldomly straight in clinical practice [17]. Many structural mechanics MOO studies on stent design aimed to improve the structural behavior of stents while minimizing their thickness. Although lower thickness was associated with higher rates of radial recoil [141], some studies achieved decreased dogboning, foreshortening and radial recoil by reducing the strut and connector width [141, 142]. Furthermore, MOO was applied to enhance the structural response of biodegradable polymeric and metallic coronary stents [134, 143, 144].

Both rectangular and circular cross‐section strut stents have been optimized for hemodynamic and structural mechanics objectives, with adverse hemodynamic performance of mechanically superior thicker struts being successfully offset by a larger element spacing [100]. When considering hemodynamics indicators only, semi‐circular struts were optimized to minimize recirculation areas and maximize fluid reattachment length [138]. Additionally, optimization of ESS was achieved by determining the optimal strut number for a given stent length [139], while the optimal number of circumferential elements was shown to depend on the intrastrut angle [138].

Incorporating drug metrics, such as volume average drug and uniformity of drug distribution, provided valuable insights, including the impact of stent design on drug distribution after deployment. Notably, a wider strut is beneficial for drug delivery but detrimental to other objectives, such as arterial stress [140]. MOO allowed the exploration and enhancement of the drug release kinetics of paclitaxel and sirolimus‐eluting stents, leading to proposed recommendations for improved drug delivery [101].

### 4.2 Consideration of Two‐Stenting Techniques

Studies on two‐stenting techniques are limited and are primarily based upon computationally idealized models without plaque that incorporate a three‐layered arterial wall and focus on procedural aspects (Table 2). A complete and validated platform to simulate one‐ and two‐stenting techniques in patient‐specific coronary bifurcations achieved accurate replication of the corresponding clinical case for stent deployment using PSB, TAP, and culotte techniques [132]. Structural mechanics and hemodynamic aspects were explored for DKC and nano‐crush, culotte, and TAP techniques. For instance, POT had a positive hemodynamic effect on the nano‐crush and T strategies, yet a neutral and even negative impact on culotte and DKC, respectively [145]. Distal balloon positioning for the final POT showed improved hemodynamics in both DKC and nano‐crush [146].

**TABLE 2:** Comparison of computational and bench testing studies investigating two‐stenting techniques in coronary artery bifurcations.

Focus	Type	Stenting technique	Coronary model				Reference
			Geometry (n)	Wall	Plaque presence	Plaque material	
Tryton stent positioning	SMFEA	Tryton‐culotte	I (1)	Three layers, isotropic hyperelastic	No	—	Chiastra et al. [87]
Tryton stent rotational positioning	SMFEA	Tryton‐culotte	I (1)	Three layers, isotropic hyperelastic	No	—	Grundeken et al. [114]
Novel bifurcation‐dedicated stent design	SMFEA	Novel technique	I (1)	Three layers, isotropic hyperelastic	Yes	Isotropic hyperelastic	Arokiaraj et al. [66]
POT effect on hemodynamics	CFD	DKC + T + culotte + nano‐crush	I (1)	Not provided	Yes	Geometric stenosis	Rigatelli et al. [145]
POT balloon positions	CFD	Nano‐crush + DKC	PS (8)	Three layers, isotropic hyperelastic	Yes	Not provided	Zuin et al. [146]
Deployment comparison with and without the Tryton stent	SMFEA + CFD	PSB + culotte + Tryton‐culotte	I (1)	Three layers, homogeneous isotropic hyperelastic	No	—	Morlacchi et al. [147]
Structural and fluid dynamic analysis	SMFEA + CFD	Simultaneous kissing stent	I (1)	Not provided	No	—	Morris et al. [67]
Drug‐eluting analysis	SMFEA + CFD	PSB + culotte + Tryton‐culotte	I (1)	One layer, constant thickness of 0.9 mm	No	—	Cutri et al. [148]
SB diameter and bifurcation angle	BT + CFD	DK‐nano‐crush	I (2)	Not provided	No	—	Morris et al. [96]
Comparison between DKC and TAP techniques	BT + CFD	DKC + TAP	I (1)	Not provided	No	—	Paradies et al. [149]
Comparison between crush, culotte and T/TAP	BT + CFD	Crush + culotte + T/TAP	I (2)	Not provided	No	—	Foin et al. [94]
Bifurcation angle and flow disturbances	BT + SMFEA + CFD	PSB + crush + culotte + T	I (1)	One layer, linear elastic	No	No	Raben et al. [150]
Novel computational platform for PS stenting planning	BT + SMFEA + CFD	PSB + TAP + culotte	PS	Isotropic hyper‐elastic	Yes	Isotropic hyperelastic with plaque reconstruction from OCT	Zhao et al. [132]
Experimental investigation of stented hemodynamics	BT	PSB + culotte + crush	I (4)	PDMS–Sylgard 184	No	—	Brindise et al. [115]

Abbreviations: BT: bench testing; CFD: computational fluid dynamics; DKC: double‐kissing crush; I: idealized; OCT: optical coherence tomography; POT: proximal optimization technique; PS: patient‐specific; PSB: provisional side branch; SMFEA: structural mechanics finite element analysis; TAP: T‐and‐protrusion.

An effective variation to the classic DKC with nano‐protrusion was proposed to minimize flow alteration by the stent struts [96]. Additionally, a novel implant strategy involving a cobalt‐chrome design in the main branch with a central part made of nitinol and a different geometry was introduced to facilitate the SB stenting [66]. This technique demonstrated the preservation of the SB and lower stresses within the artery wall and stent compared to other stenting techniques [66].

The role of patient‐specific characteristics was considered in only one study on the impact of the bifurcation angle on crush, culotte, and T. The findings revealed that wider angles were associated with greater areas of slow flow and recirculation [150]. Compared with single stenting, the use of crush, culotte, and T resulted in an increased metal‐to‐artery ratio and the formation of a metallic neocarina, which overall altered the local hemodynamics [150]. When considering all the techniques together, the nano‐crush and T techniques demonstrated superior flow conditions, while DKC and culotte showed the least favorable hemodynamic results [145]. PSB and culotte techniques were compared with respect to the final drug distribution [148], demonstrating that two DES significantly improved the effectiveness of the drug delivery. The study also highlighted that the different stenting techniques result in varied contact surfaces between the stent and the artery, which strongly influences drug release. The culotte technique exhibits a wide region of stent overlap, leading to considerable drug loss in the bloodstream, whereas one‐stent cases show minimal drug loss due to better strut apposition [148]. A single study explored the simultaneous kissing stent technique [67], whereby two balloon‐expandable stents are deployed at the same time in the MV and SB. Here, structural mechanics FEA revealed minimal stent distortion and moderate arterial stresses, and transient CFD showed predominantly undisturbed blood flow with some recirculation areas [67]. In contrast, the simultaneous kissing stent technique is not recommended by the European Bifurcation Club due to the unpredictability observed in clinical practice, including stent distortion, arterial scaffolding, and the formation of a double‐layer neocarina [44].

The performance of the bifurcation‐dedicated Tryton stent has been studied before as the only dedicated bifurcation stent on the market [87, 114, 147, 148, 151]. Its design is characterized by an unconventional geometry that facilitates the stenting of the SB through a culotte‐like strategy. The recommended positioning achieved the highest SB diameter and lowest arterial stress compared to a more distal or proximal positioning [87]. Rotational positioning did not significantly influence the stent performance, but rewiring through the wrong panel increased arterial damage [114]. The Tryton‐based culotte technique had a reduced metal‐to‐artery ratio and, as a result, a better hemodynamic performance than a standard culotte [147]. Moreover, the Tryton design demonstrated superior stent apposition and more effective drug delivery compared with other techniques, minimizing drug losses resulting from strut overlapping [148].

To date, bench testing is the most common way to achieve stent deployment when evaluating two‐stenting techniques. Using an in vitro setup, the micro‐CT stented phantom reconstructions revealed that crush and culotte techniques improved scaffolding but resulted in higher levels of malapposed struts compared to TAP [94]. The crush and culotte techniques generated a greater amount of metal due to the formation of a metallic neo‐carina and multiple layers of struts against the lateral wall, damaging the polymeric coating [94]. Notably, when considering the crush variation DKC, TAP, and DKC were found to achieve similar malapposition rates and SB ostium clearance. Particle image velocimetry considerations showed that culotte led to higher TAESS and smaller areas of low and recirculating flow than crush [115].

## 5 Discussion and Outlook

The optimal treatment of coronary bifurcation lesions continues to evolve. Clinical trials have proved the effectiveness of the PSB technique in addressing simple lesions, whereas two‐stenting techniques have shown better outcomes for cases involving complex diseased vasculature [32]. Bench testing has had a primary role in analyzing stenting techniques, and in the past two decades, both structural mechanics and hemodynamic computational simulations have offered the ability to evaluate quantitative insights that would not have been achievable through in vitro or in vivo experiments alone, such as arterial injury or ESS.

This review highlights the extensive use of investigations to comprehend the dynamics of the coronary bifurcated artery, stent, and blood flow during and after deployment. Their findings have contributed to the improvement of stenting techniques, such as the determination of the optimal balloon positioning, the importance of SB rewiring positioning, or the superiority of POT over the KBI [46], resulting in consensus guidelines by interventional cardiologists, such as those of the European Bifurcation Club [30, 152].

We discussed relevant methods for the investigation of stenting techniques to delineate which may be best suited for which patient‐specific scenario. Key challenges with the use of these methods persist. Specifically, the fidelity of the model, physical or virtual, plays a crucial role in the reliability of the results. The account of plaque shape and composition and its variability within and between patients remains an ongoing bottleneck in the field. In fact, most studies neglected plaque altogether, forming a persisting drawback in the literature to date, despite the demonstrated significant impact of plaques. In addition, most models consider planar bifurcations, disregarding the three‐dimensionally curved nature of coronaries, which also have been shown to impose important alternations and thus mitigate the applicability of many current observations. To date, these challenges can mainly be attributed to the lack of large computational resources. In fact, when evaluating scenarios involving multiple stent implantations, where numerous struts interact with the surrounding environment, the analysis demands millions of discretized elements. These elements are frequently computed over finely spaced time steps to achieve reliable results. Consequently, there is a significant rise in computational time, which is amplified with each additional aspect integrated into the model, such as multi‐material properties of plaques, vessel characteristics, or patient‐specific variations. In vitro testing is limited by its high costs, particularly in achieving a large number of phantoms with softer material properties and plaque.

Drug‐eluting balloons (DEB) have emerged as a promising treatment for ISR, delivering anti‐proliferative drugs directly to the lesion site without adding another stent layer. This approach has shown effectiveness, particularly in smaller vessels or in cases where additional stents may induce further restenosis [153]. Recent data demonstrated lower major adverse cardiac events at 12 months with hybrid strategies combining DES in the MV and DEB in the SB for non‐complex, true bifurcation lesions [154]. Further research is needed to explore fully stent‐free interventions for bifurcation lesions. Computational modeling can enhance our understanding of DEB performance in complex bifurcation lesions, offering insights into its interactions with the arterial wall and blood flow dynamics.

Surrogate models have elevated computational testing and, as a result, offer significant advantages for this field. By accurately approximating the relationship between variables and objectives, surrogate models notably decrease the number of simulations required to evaluate a wide range of scenarios. This reduction in computational burden is particularly beneficial for MOO, which has great potential but has been applied commonly to stent design optimization and only once to stent strategy optimization to date due to its complexity. Through the literature, we highlighted MOO's potential in this context, which offers opportunities to explore implant techniques with their multitude of influencing factors and potentially competing trade‐offs, thus advancing the field toward a more personalized, granular direction. Indeed, MOO can help reduce the time required to investigate numerous scenarios simultaneously, but its effectiveness is still partially dependent on the available resources.

As numerical simulations continue to advance in speed, cost‐effectiveness, and accessibility; this technique may even be applied to devise optimal interventional strategies tailored to each patient‐specific case and may be validated with bench efforts and tailored platforms.

## Author Contributions

**Andrea Colombo and Susann Beier:** conceptualization. **Andrea Colombo:** literature search. **Andrea Colombo:** writing – original draft preparation. **Susann Beier, Claudio Chiastra, Diego Gallo, Tapabrata Ray, Nigel Jepson, Poay Huan Loh, Socrates Dokos, Mingzi Zhang, Hamed Keramati, Dario Carbonaro, and Francesco Migliavacca:** writing – critical review of the draft.

## Ethics Statement

The authors have nothing to report.

## Conflicts of Interest

The authors declare no conflicts of interest.

## Supporting information


**Data S1.** Supporting Information.

## Data Availability

Data sharing not applicable to this article as no datasets were generated or analysed during the current study.
