# Supplementary material for: Advancements in Coronary Bifurcation Stenting Techniques: Insights From Computational and Bench Testing Studies
Source: Int J Numer Method Biomed Eng. 2025 Mar 14;41(3):e70000. doi: 10.1002/cnm.70000 (PMC11909422; doi:10.1002/cnm.70000)
Supplement: Supplementary file 1 — Data S1. Supporting Information. [file CNM-41-e70000-s001.docx]

**Supplementary Material**


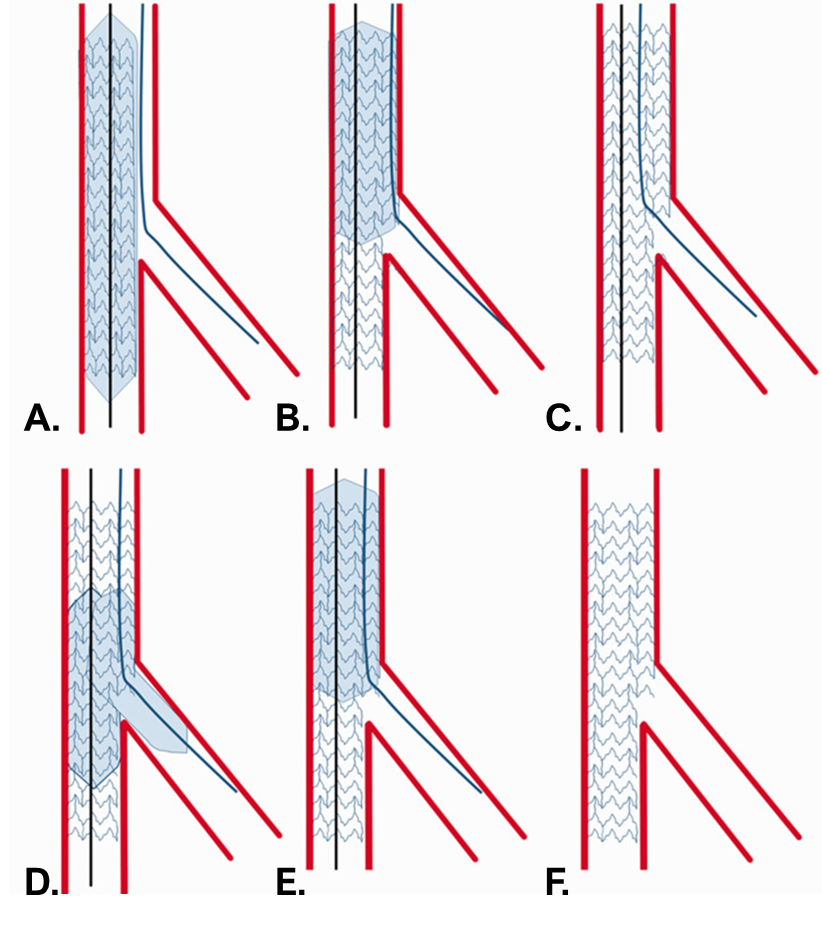


**Fig. S1** Provisional side branch procedural steps. **A**. Main vessel stenting **B**. Proximal optimization technique **C**. Side branch rewiring **D**. Kissing balloon inflation **E**. Proximal optimization technique **F**. Final configuration. Reproduced with permission from Raphael et al. [1].


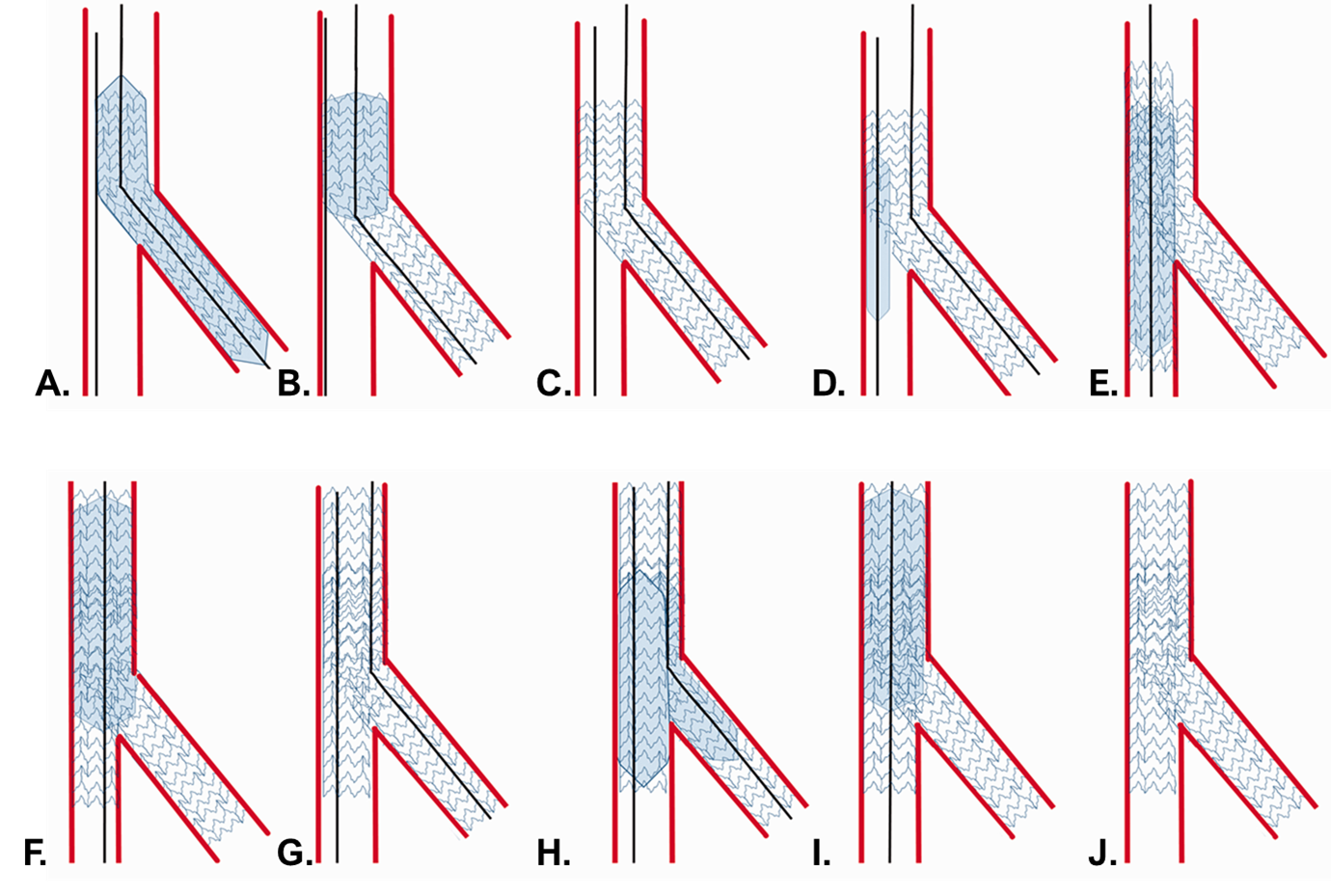


**Fig. S2** Culotte procedural steps. **A**. Side branch stenting with protrusion **B**. Proximal optimization technique **C**. Rewiring **D**. Opening of the stent struts in the main vessel **E**. Main branch stenting **F**. Proximal optimization technique **G**. Side branch rewiring **H**. Kissing balloon inflation **I**. Proximal optimization technique **J**. Final configuration. Reproduced with permission from Raphael et al. [1].


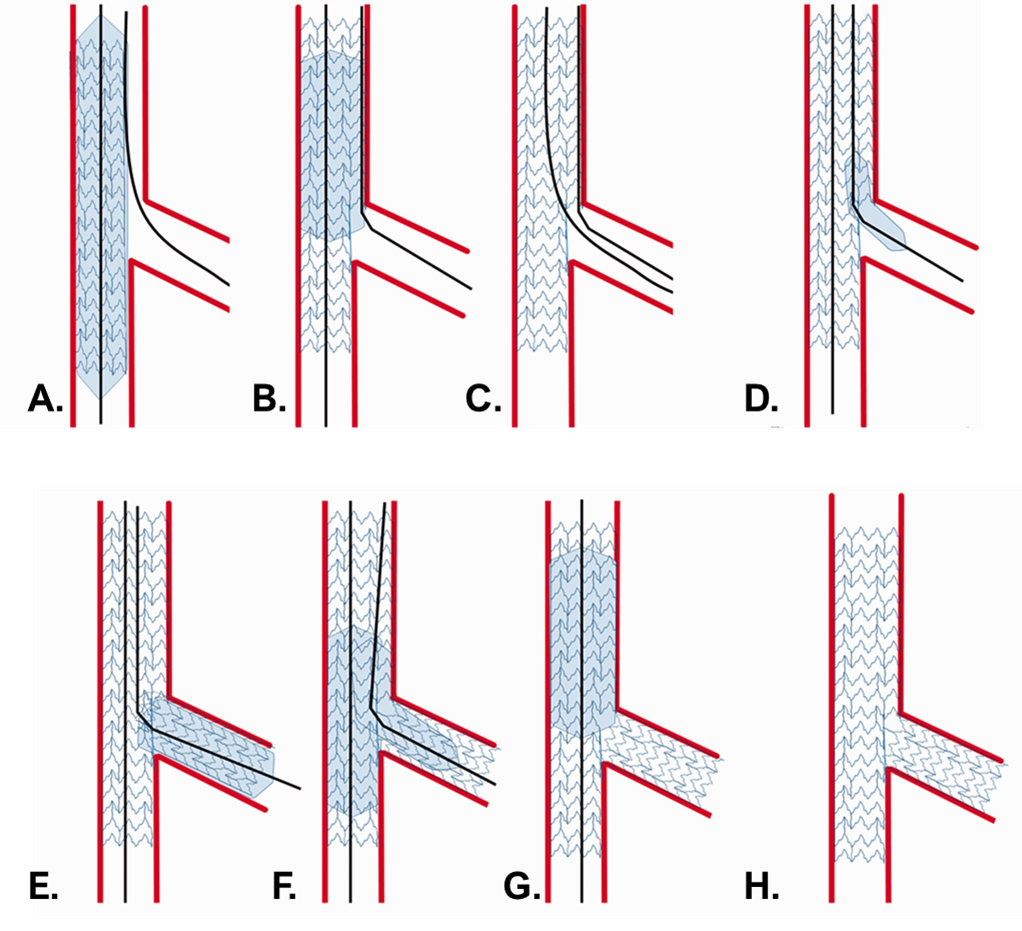


**Fig. S3** T/TAP procedural steps. **A**. Main branch stenting **B**. Proximal optimization technique **C**. Side branch rewiring **D**. Opening of the stent struts with a small balloon **E**. Side branch stenting with minimal protrusion **F**. Kissing balloon inflation **G**. Proximal optimization technique **H**. Final configuration. Reproduced with permission from Raphael et al. [1].


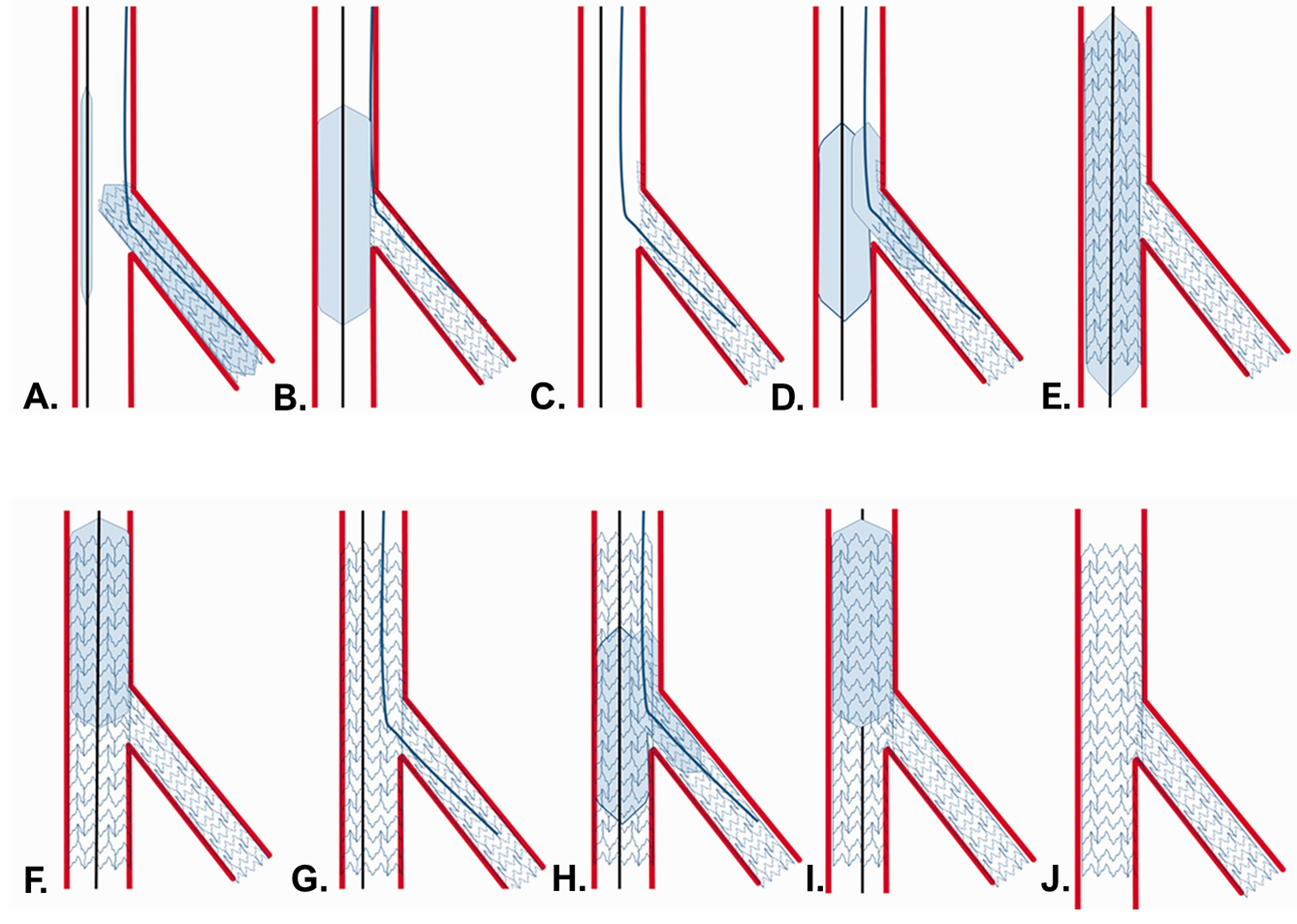


**Fig. S4** Double-Kissing Crush procedural steps. **A**. Side branch stenting with 2/3mm of protrusion **B**. Balloon crush **C**. Side branch rewiring **D**. Kissing balloon inflation **E**. Main branch stenting **F**. Proximal optimization technique **G**. Side branch rewiring **H**. Kissing balloon inflation **I**. Proximal optimization technique **K**. Final configuration. Reproduced with permission from Raphael et al. [1].

**Table S5** Review of works that investigated PSB, indicating which aspects were considered, the main results, and the type of analysis. BT: Bench Testing; CFD: Computational Fluid Dynamics; MB: Main Branch; POT: Proximal Optimization Technique; PSB: Provisional Side Branch; SB: Side Branch; SMFEA: Structural Mechanics Finite Element Analysis.

| **Implant technique** | | | |
| --- | --- | --- | --- |
| Balloon | Diameter | Larger balloon diameters resulted in a larger stent opening towards the SB but also in higher stresses in the SB wall [2]. | SMFEA |
|  | Length | SB access was worsened by longer balloons but not impacted by shorter ones [3]. | SMFEA |
|  |  | An optimal ratio between balloon and stent length could optimize dog-boning effects [4] | SMFEA |
|  | Shape | The use of a tapered balloon during kissing balloon inflation could reduce complications while maintaining the procedure's beneficial effects [5]. | SMFEA+CFD |
|  | Positioning | Non-central positioning between the stent and the balloon resulted in an unsymmetric deployment and worse structural behavior [4]. | SMFEA |
|  | Folding | Five-fold balloons have a smaller diameter after deflation compared to tri-fold ones, making them easier to retrieve [4]. | SMFEA |
|  | Thickness | A non-uniform balloon thickness leads to a non-uniform stent expansion pattern [4]. | SMFEA |
| SB access | Cell positioning | A distal SB access led to better hemodynamic results with respect to a proximal one for kissing balloon inflation [6]. | SMFEA+CFD |
|  |  | Proximal rewiring obtained higher ostial stenosis, rate of malapposed struts, and area with high WSS [7]. | BT+CFD |
|  |  | Central cell rewiring for kissing balloon inflation achieved better SB ostium area and lower stresses in both the stent and the artery with respect to proximal and distal positionings [8]. | SMFEA |
| Final kissing balloon | Effect compared to POT | Final kissing balloon led to higher arterial stresses and stent plastic strains [8]. | SMFEA |
|  |  | Final kissing balloon was seen to be less effective than POT [9]. | BT+  SMFEA+CFD |
|  | Effect compared to only MB stenting | A greater strut-free ostium area but higher stresses were observed with final kissing balloon [5]. | SMFEA+CFD |
|  | Inflation | Non-simultaneous inflation of the two balloons showed lower ostial stenosis and elliptical deformation in the proximal vessel, with same rates of malapposition with respect to a simultaneous one [10]. | SMFEA |
| POT | Effect compared to final kissing balloon | POT led to lower arterial stresses and stent plastic strains [8]. | SMFEA |
|  | Positioning | Central POT resulted in lower malapposition rates and higher post-expansion diameters with respect to proximal and distal positioning [11]. | SMFEA |
| Stent | Overlapping | High arterial and stent stress were found in the overlapping region [12]. | SMFEA |
| **Stent design** | | | |
| Strut | Thickness | Stents with large strut thickness led to a stiffer device that induced higher stresses [13]. | SMFEA |
| Design | Open/closed cell | Open cell stents enlarge wider after angioplasty with respect to closed cell devices [2]. | SMFEA |
|  |  | Open cell were seen to have a more flexible design that induced less stress in the artery [13]. | SMFEA |
|  | Commercial design | No statistical differences were found between commercial designs [7, 10]. | BT+CFD; SMFEA |
| Size | Oversizing | Stent oversizing led to less malapposition but higher arterial stress [14] | SMFEA+CFD |
| **Patient-specific characteristics** | | | |
| Artery | Curvature | Compliance mismatch between artery and device increases wall stress at the discontinuity [13]. | SMFEA |
|  | Bifurcation Angle | The bifurcation angle did not impact SB access [3]. | SMFEA |
| Plaque | Material | The presence of plaque material, especially calcified ones, significantly affects SB access. While fibrous or lipid plaques have minimal impact on lumen dimensions, calcified plaques lead to significant variations in lumen area [3]. | SMFEA |
|  | Presence | The presence of the plaque presented worse mechanical and hemodynamic indicators compared to a plaque-free coronary [15]. | SMFEA+CFD |

**References**

[1] Raphael, C.E. & O’Kane, P.D. 2021 Contemporary approaches to bifurcation stenting. *JRSM Cardiovascular Disease* **10**, 2048004021992190.

[2] Mortier, P., De Beule, M., Van Loo, D., Verhegghe, B. & Verdonck, P. 2009 Finite element analysis of side branch access during bifurcation stenting. *Medical Engineering & Physics* **31**, 434-440. (doi:<https://doi.org/10.1016/j.medengphy.2008.11.013>).

[3] Iannaccone, F., Chiastra, C., Antonios, K., Francesco, M., Frank, J.H.G., Patrick, S., Peter, M., Benedict, V., Gabriele, D., Matthieu De, B., et al. 2017 Impact of plaque type and side branch geometry on side branch compromise after provisional stent implantation: a simulation study. *EuroIntervention* **13**, e236-e245. (doi:10.4244/EIJ-D-16-00498).

[4] Rahinj, G.B., Chauhan, H.S., Sirivella, M.L., Satyanarayana, M.V. & Ramanan, L. 2022 Numerical Analysis for Non-Uniformity of Balloon-Expandable Stent Deployment Driven by Dogboning and Foreshortening. *Cardiovascular Engineering and Technology* **13**, 247-264. (doi:10.1007/s13239-021-00573-4).

[5] Morlacchi, S., Chiastra, C., Gastaldi, D., Pennati, G., Dubini, G. & Migliavacca, F. 2011 Sequential Structural and Fluid Dynamic Numerical Simulations of a Stented Bifurcated Coronary Artery. *Journal of Biomechanical Engineering* **133**. (doi:10.1115/1.4005476).

[6] Chiastra, C., Morlacchi, S., Pereira, S., Dubini, G. & Migliavacca, F. 2012 Computational fluid dynamics of stented coronary bifurcations studied with a hybrid discretization method. *European Journal of Mechanics - B/Fluids* **35**, 76–84. (doi:10.1016/j.euromechflu.2012.01.011).

[7] Foin, N., Torii, R., Alegria, E., Sen, S., Petraco, R., Nijjer, S., Ghione, M., Davies, J.E. & Di Mario, C. 2013 Location of side branch access critically affects results in bifurcation stenting: Insights from bench modeling and computational flow simulation. *International Journal of Cardiology* **168**, 3623-3628. (doi:<https://doi.org/10.1016/j.ijcard.2013.05.036>).

[8] Gastaldi, D., Morlacchi, S., Nichetti, R., Capelli, C., Dubini, G., Petrini, L. & Migliavacca, F. 2010 Modelling of the provisional side-branch stenting approach for the treatment of atherosclerotic coronary bifurcations: effects of stent positioning. *Biomechanics and Modeling in Mechanobiology* **9**, 551-561. (doi:10.1007/s10237-010-0196-8).

[9] Foin, N., Torii, R., Mortier, P., De Beule, M., Viceconte, N., Chan, P.H., Davies, J.E., Xu, X.Y., Krams, R. & Di Mario, C. 2012 Kissing Balloon or Sequential Dilation of the Side Branch and Main Vessel for Provisional Stenting of Bifurcations: Lessons From Micro-Computed Tomography and Computational Simulations. *JACC: Cardiovascular Interventions* **5**, 47-56. (doi:<https://doi.org/10.1016/j.jcin.2011.08.019>).

[10] Mortier, P., Hikichi, Y., Foin, N., De Santis, G., Segers, P., Verhegghe, B. & De Beule, M. 2014 Provisional Stenting of Coronary Bifurcations: Insights Into Final Kissing Balloon Post-Dilation and Stent Design by Computational Modeling. *JACC: Cardiovascular Interventions* **7**, 325-333. (doi:<https://doi.org/10.1016/j.jcin.2013.09.012>).

[11] Rigatelli, G., Zuin, M., Chiastra, C. & Burzotta, F. 2020 Biomechanical Evaluation of Different Balloon Positions for Proximal Optimization Technique in Left Main Bifurcation Stenting. *Cardiovasc Revasc Med* **21**, 1533-1538. (doi:10.1016/j.carrev.2020.05.028).

[12] Morlacchi, S., Colleoni, S.G., Cárdenes, R., Chiastra, C., Diez, J.L., Larrabide, I. & Migliavacca, F. 2013 Patient-specific simulations of stenting procedures in coronary bifurcations: Two clinical cases. *Medical Engineering & Physics* **35**, 1272-1281. (doi:<https://doi.org/10.1016/j.medengphy.2013.01.007>).

[13] Mortier, P., Holzapfel, G.A., De Beule, M., Van Loo, D., Taeymans, Y., Segers, P., Verdonck, P. & Verhegghe, B. 2010 A Novel Simulation Strategy for Stent Insertion and Deployment in Curved Coronary Bifurcations: Comparison of Three Drug-Eluting Stents. *Annals of Biomedical Engineering* **38**, 88-99. (doi:10.1007/s10439-009-9836-5).

[14] Mortier, P., Wentzel, J.J., De Santis, G., Chiastra, C., Migliavacca, F., De Beule, M., Louvard, Y. & Dubini, G.A. 2015 Patient-specific computer modelling of coronary bifurcation stenting: the John Doe programme. *EuroIntervention* **11**, V35-V39. (doi:10.4244/EIJV11SVA8).

[15] Chen, H.Y., Chatzizisis, Y.S., Louvard, Y. & Kassab, G.S. 2020 Computational Simulations of Provisional Stenting of a Diseased Coronary Artery Bifurcation Model. *Sci Rep* **10**, 9667. (doi:10.1038/s41598-020-66777-1).
